# *C. elegans* LIM-8 localizes to aligned actin filaments in the embryonic epidermis but is not important during embryonic elongation

**DOI:** 10.17912/micropub.biology.002414

**Published:** 2026-10-26

**Authors:** Adelle Markle, Jeff Hardin

**Affiliations:** 1 Cellular and Molecular Biology Program, UW–Madison, Madison, WI, United States; 2 Department of Biology, UW–Madison, Madison, WI, United States

## Abstract

During embryonic elongation in *C. elegans* multiple actin-binding proteins cooperate to regulate cell shape changes. We investigated whether LIM-8, a LIM domain-containing protein expressed in the elongating epidermis, contributes to this process. We endogenously tagged all isoforms of LIM-8; LIM-8::mNeonGreen localizes to aligned actin filaments in the embryonic epidermis during elongation, in addition to several adult tissues. LIM-8 is not essential for embryonic elongation and we were unable to identify functional interactions with *hmp-1* /α-catenin, *sma-1* /β _H_ -spectrin, or other LIM domain containing proteins, suggesting that, despite its striking localization, LIM-8 does not make major contributions to actin regulation during embryonic elongation.

**Figure 1. LIM-8 is expressed in the epidermis during embryonic elongation as well as other adult tissues but does not make major contributions to embryonic elongation:** A: Schematic of the *lim-8* locus showing tagging strategy. Black boxes: coding exons. White boxes: UTR. Green boxes: FLAG, linker, and mNeonGreen-encoding exons. B-D: Embryos showing LIM-8::mNG expression at comma stage, two-fold, and late elongation. Images are maximum-intensity Z projections of 0.9 µm stacks. Scale bars = 10 µm. E: LIM-8::mNG colocalizes with LifeAct on circumferential filament bundles (CFBs) and along adherens junctions. Images are maximum-intensity Z projections of 0.9 µm stacks. White arrow: CFBs. White arrowhead: seam cell actin mesh. Black arrow: adherens junction at a seam-non-seam boundary. Scale bar = 10 µm. F: LIM-8::mNG expression in the pharynx of an adult worm. Image is a maximum intensity Z projection of a 4.5 µm stack. White arrow: anterior bulb. White arrowhead: terminal bulb. Scale bar = 10 µm. G: LIM-8::mNG expression in the uterus of an adult worm merged with the corresponding DIC image. Scale bar = 20 µm. H: LIM-8::mNG expression in the rectal and posterior phasmid systems. Image is a maximum-intensity Z projection of a 9.5 µm stack. Due to the strength of the signal in the phasmid cells, this image was acquired at lower laser power than other images. White arrows: phasmid system. White arrowhead: rectal system. Scale bar = 10 µm. I: LIM-8::mNG expression in an occupied spermatheca. LIM-8 signal is not specific to actin bundles but does accumulate at cell-cell junctions in a more apical view (I'). Images are maximum-intensity Z projections of 1.5 µm stacks, with I' immediately apical to I. Scale bar = 10 µm. J: Lethality counts do not reveal any role for *lim-8* in sensitized backgrounds. N represents the number of adult worms whose progeny was scored. ns, not significant (P > 0.05 in all cases).

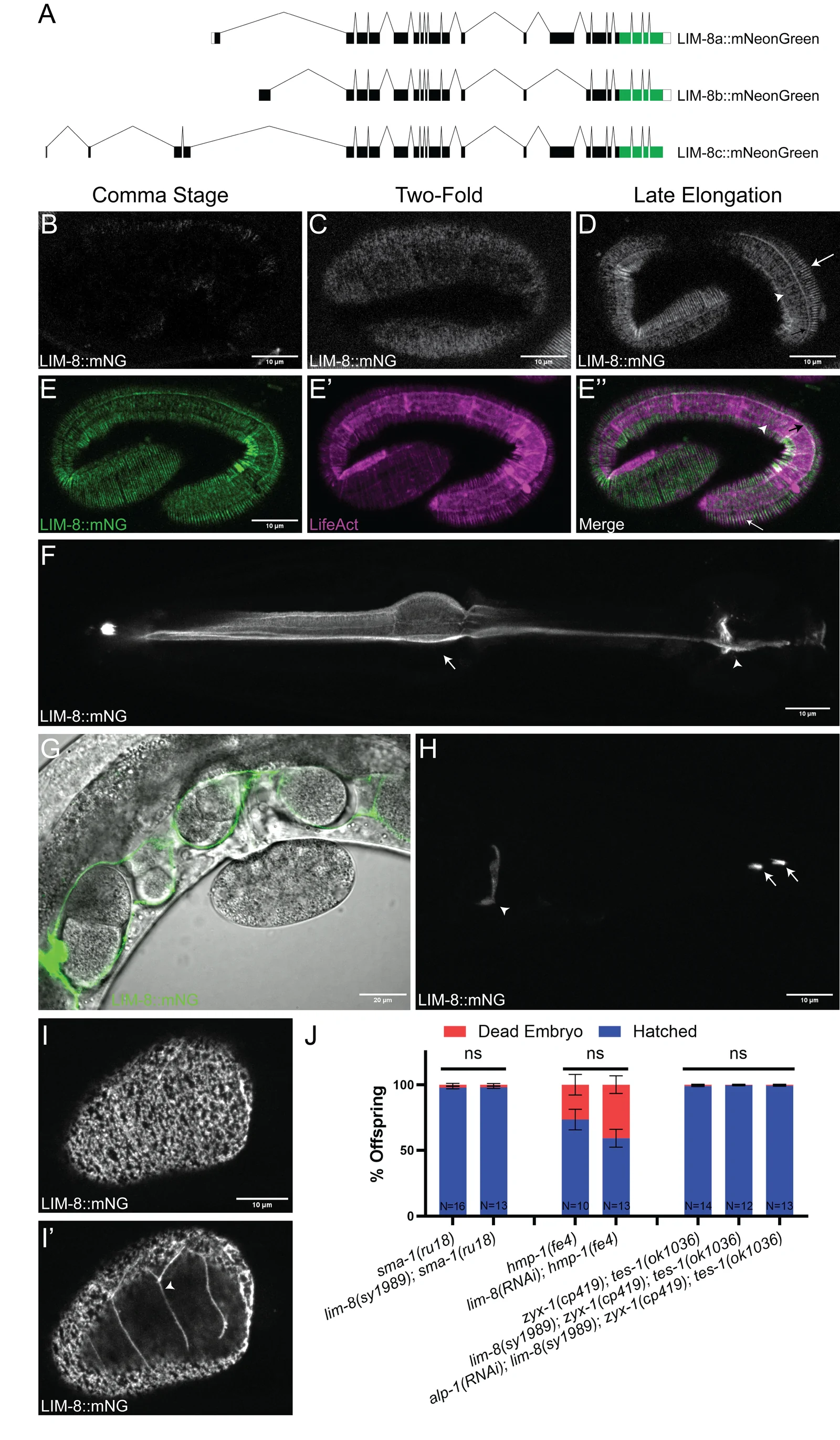



## Description

Embryonic elongation of the *C. elegans* embryo is a powerful system for exploring the actin regulatory mechanisms that facilitate coordinated cell shape change. During embryonic elongation, the *C. elegans* embryo increases in length four-fold while reducing its diameter three-fold; this dramatic morphological change is accomplished by actomyosin-mediated epidermal cell shape changes, without any contribution from cell division or migration (reviewed in Labouesse et al., 2026). Proteins containing LIM domains are known to bind actin filaments under tension and have been implicated in repair of strained actin bundles (Smith et al., 2010; Smith et al., 2014; Sun et al., 2020). In *C. elegans* , LIM domain proteins have been investigated in striated body wall muscle (Lecroisey et al., 2013; Lecroisey et al., 2008; Qadota et al., 2007), synapses (Luo et al., 2014), and gastrulation (Slabodnick et al., 2023). We recently showed that two LIM domain containing repeat (LCR) proteins, ZYX-1 and TES-1 , play roles at cell-cell junctions in elongating *C. elegans* embryos (Lynch et al., 2022), and so we sought to examine other *C. elegans* LIM domain containing proteins in the context of embryonic elongation. One such protein that has not been characterized during elongation is LIM-8 , despite available single cell RNAseq data indicating it is expressed in the epidermis during elongation (Packer et al., 2019).

We endogenously tagged all known LIM-8 isoforms with mNeonGreen using CRISPR/Cas9 genome editing technology ( Fig. 1A ). LIM-8 ::mNG signal is first visible during early elongation, though it remains dim until the two-fold stage ( Fig. 1B, 1C). During the second, muscle contraction-dependent phase of elongation, LIM-8 ::mNG signal is bright and localized to circumferential F-actin filament bundles (CFBs; Priess and Hirsh, 1986) in dorsal and ventral epidermal cells, the mesh-like actin network in seam epidermal cells (Costa et al., 1997; Gally et al., 2009), and proximal actin networks associated with adherens junctions (Costa et al., 1998; Fig. 1D, 1E). These observations are consistent with scRNA seq data for *lim-8* from a published dataset, which shows transcriptional expression of *lim-8* in AB- and C-derived epidermis and seam lineages 390-510 min after the first cell division (Packer et al., 2019). However, it is unclear from these data and ours whether the change in expression results from uniform expression during this time window, from increasingly specific localization, or both. Future experiments will be required to adjudicate these possibilities. During the muscle contraction-dependent phase of elongation, the LIM-8 ::mNG knock-in may have utility as an endogenously encoded reporter of F-actin organization, particularly as an alternative to LifeAct (Havrylenko et al., 2015) or the F-actin-binding fragment of the spectraplakin VAB-10 (Gally et al., 2009) for assessing the organization and alignment of CFBs in the embryonic epidermis.

In adult worms, LIM-8 ::mNG signal is visible in the pharynx, as expected from previously reported promoter::GFP fusion experiments (Qadota et al., 2007; Fig. 1F ). LIM-8 ::mNG is also expressed in the uterus, the rectal and phasmid systems, and the spermatheca ( Fig. 1G, 1H, 1I). Unlike in the epidermis of elongating embryos, LIM-8 in the spermatheca does not accumulate on aligned actin filaments but instead has a speckled appearance and localizes basal to adherens junctions ( Fig. 1I ). This localization pattern was observed in both occupied and unoccupied spermathecae, suggesting that the tension state of the actin filaments in the spermatheca does not determine whether LIM-8 is recruited. In the spermatheca, LIM-8 also localizes to apical adherens junctions ( Fig. 1I '). While LIM-8 has been shown by antibody staining to localize to the M line in adult body wall muscle (Qadota et al., 2007), we were unable to detect LIM-8 ::mNG in body wall muscle under our imaging conditions.

While *lim-8* loss of function alone is not associated with overt phenotypes (Qadota et al., 2007), the strong actin localization of LIM-8 during elongation, combined with previously identified roles for LIM-domain containing proteins TES-1 and ZYX-1 using sensitized backgrounds (Lynch et al., 2022), led us to consider whether LIM-8 might be functionally redundant with other actin-binding proteins during elongation. Since both *tes-1* and *zyx-1* loss of function enhance the lethality of the hypomorphic α-catenin mutant *hmp-1 ( fe4 )* , we tested whether *lim-8* might also be a *hmp-1 ( fe4 )* enhancer. We observed no significant difference in embryonic lethality between offspring of *hmp-1 ( fe4 )* mothers and *hmp-1 ( fe4 )* mothers injected with *lim-8* dsRNA ( Fig. 1J ). We also tested whether a STOP-IN null allele (Wang et al., 2018) for *lim-8* would synergize with mutant alleles for other genes involved in elongation. The β _H_ -spectrin *sma-1* is essential for efficient elongation, and loss of *sma-1* function, while not lethal, results in hatchlings that reach just over two-fold, rather than complete four-fold elongation (McKeown et al., 1998; Praitis et al., 2005). A *lim-8 ; sma-1* double null line did not show any embryonic lethality, and no obvious additional morphological defects were observed on plates ( Fig. 1J ). To ask whether loss of LIM-8 might synergize with other LIM domain-containing proteins during elongation, we crossed the *lim-8* null allele with *tes-1* and *zyx-1* null alleles. Since the resulting line likewise did not display embryonic lethality, we considered whether another LIM domain containing protein, ALP-1 , might be supporting elongation in this background through functional redundancy ( Fig. 1J ). As the existing Ahringer library clone targeted to *alp-1* covers sequence unique to the d isoform and the clone used to knock down *alp-1* expression in previous studies is targeted to *T11B7.5* , a gene found in an intron of *alp-1* , and therefore is not specific to *alp* -1 (McKeown et al., 2006), we generated a new RNAi clone targeting all known isoforms of *alp-1* but only sequences specific to that gene. Injection of *alp-1* dsRNA generated using this clone into the *lim-8 (sy1989); tes-1 ( ok1036 ); zyx-1 ( cp419 )* line did not result in any embryonic lethality ( Fig. 1J ).

From these results, we conclude that LIM-8 does not make major contributions to *C. elegans* embryonic elongation. Any role it may play is dispensable for proper elongation; it is possible that additional proteins act redundantly with LIM-8 that we did not address in this study. Nevertheless, the differences in LIM-8 localization between the epidermis and the spermatheca suggest that LIM-8 may be a useful probe for interrogating the mechanisms regulating the association of LIM domain-containing proteins with actin filaments, which may be of biological relevance in other contexts.

## Methods

*Strains* Hermaphrodite *C. elegans* were maintained at 20°C on nematode growth media (NGM) plates seeded with *E. coli* strain OP50 according to standard protocols. N2 Bristol was used as wildtype (Brenner, 1974).

Strains used:

N2 : *Wildtype,* from *C. elegans* Genetics Center (CGC)

SU1073 : *zyx-1 ( cp419 )II; tes-1 ( ok1036 )IV* from (Lynch et al., 2022)

PS9957 : *lim-8 ( sy1989 ) III* from CGC (Wang et al., 2018)

SU1164 : *zyx-1 ( cp419 ) II; lim-8 ( sy1989 ) III; tes-1 ( ok1036 ) IV* from this study

SU1168 : *lim-8 ( jc116 [ lim-8 ::FLAG::mNG+LoxP]) III 2xOC* from this study

AZ30 : *sma-1 ( ru18 ) V* from (Praitis et al., 2005)

SU1170 : *sma-1 ( ru18 ) V 4xOC* from this study

SU1349 : *curIs11 [plin-26::LifeAct::mCherry]; lim-8 ( jc116 [ lim-8 ::FLAG::mNG+LoxP]) III* from this study. Note: the LifeAct integrant likely resides on *LG X*

*hmp-1 ( fe4 ) V* isolated from balanced line SU287 ( *hmp-1* ( *fe4* )/ *lon-3* ( *e2175* ) V) (Lynch et al., 2012) on 6/20/2026

*Confocal imaging* Embryos were mounted on 10% agar pads in M9 buffer according to standard protocols (Hardin et al., 2022). Embryos were surrounded with OP50 during mounting to paralyze them by hypoxia (Gally et al., 2009). Confocal images were collected at 20°C using a Dragonfly 500 spinning disc confocal microscope (Oxford Instruments) mounted on a Leica DMi8 microscope equipped with a 63X glycerol immersion objective and a Zyla camera and controlled by Fusion software (Oxford Instruments) using a slice spacing of 0.3 μm. Adult worms were mounted on 9% agarose pads in M9 buffer and imaged using the same settings as for embryos, except that Z stacks were collected with a step size of 0.5 μm.

*CRISPR/Cas9 genome editing* To endogenously tag the *C. elegans* *lim-8* locus with FLAG and mNeonGreen, we used the self-excising selection cassette (SEC) method previously described by Dickinson et al. (Dickinson et al., 2015). The guide sequence was cloned into pJW1219 using blunt-end cloning to produce pARM1. A repair template for the C terminus of LIM-8 was generated using SapTrap cloning (Dickinson et al., 2018; Schwartz and Jorgensen, 2016) to assemble homology arms, linker, mNG, and self-excising cassette into the pMLS257 backbone to form pARM2. The forward primer for the 3' homology arm introduced a mutation to remove the PAM. pARM1 at 50 ng/µl and pARM2 at 15 ng/µl were injected into the gonads of adult *C. elegans* hermaphrodites along with coinjection markers pGH8 (4ng/µl), pCFJ90 (2ng/µl), and pCFJ104 (4ng/µl). Hygromycin selection for worms with the SEC, screening, and removal of SEC by heat shock were performed as described in Dickinson et al. (Dickinson et al., 2015). Oligonucleotides used for cloning, genotyping, and sequencing are as follows (Sequence complementary to *C. elegans* DNA indicated in ALL CAPS, mutation introduced to remove PAM in red):

pJW1219 F with guide sequence added: ACATTCTAGAAAGAACTTCTgtttaagagctatgctggaaacag

pJW1219 R: caagacatctcgcaataggag

pJW1219 gRNA sequencing: gtgtgaaataccgcacaga

5' homology arm forward: ggctgctcttcgtggGCTACGTCATCGTCGTTGAGG

5' homology arm reverse: gggtgctcttcgcgcGACTTTGCTCAATTGCACACGATC

3' homology arm forward: ggctgctcttcgggtTAAGCTCTAAATAGTCCAGTCgCAGAAGTTC

3' homology arm reverse: gggtgctcttcgtacGCGGTCGGTTTTGAGTTTCC

SEC sequencing reverse for repair template: ttaatgtgagttagctcactcattagg

*lim-8* knock in genotyping forward: GCTACGTCATCGTCGTTGAGG

*lim-8* knock in genotyping reverse: CCACCTCCGCCGTATAATGC

*lim-8* knock in sequencing: GGACGTGGTGCTGCAATGAT

*Cloning* Approximately 0.5kb of the *alp-1* coding region, common to all known isoforms and not sharing sequences with any other known gene, was amplified from N2 worm cDNA and cloned into the L4440 feeding RNAi vector using standard restriction cloning techniques to produce pARM4. Oligonucleotides used for cloning are as follows (Sequence complementary to *C. elegans* DNA indicated in ALL CAPS):

Forward primer for *alp-1* cDNA with HindIII restriction site added: gcaagcttGGAGTGATGGCTCGTGCAG

Reverse primer for *alp-1* cDNA with NheI restriction site added: gcgctagcCATTTGAAGGTGGTGGATTTGACGG

*RNAi and plate counts* dsRNA was generated using T7 transcription kit using a *lim-8* cDNA clone (courtesy of G. Benian) and pARM4 as templates. dsRNA was injected into L4/young adult worms at 2 µg/µl for *alp-1* and 3 µg/µl for *lim-8* . Uninjected young adults and injected worms 20-24 hours post-injection were singled and allowed to lay for 20-24 hours. For *hmp-1 ( fe4 )* and *sma-1 ( ru18 )* lines this time was extended up to 28 hours due to gonad defects in *hmp-1 ( fe4 )* and body morphology defects in both lines, which slowed egg laying. Adult worms were then removed; 20-24 hours after removal of the adult worm, the number of hatchlings and dead eggs on each plate was counted. Each experiment was performed twice.

*Statistical analysis* Percent lethality data, with the offspring of each worm considered as a biological replicate, were compared using Welch's t test (where two groups were to be compared), and one-way Brown-Forsythe and Welch ANOVA (where three groups were to be compared) to assess the significance of differences between groups using GraphPad Prism software. N values for each group are included in Figure 1J .

*scRNAseq analysis* Transcript levels for *lim-8* in epidermal cells were obtained using the summarized expression tool available in the online VisCello viewer (https://cello.shinyapps.io/celegans_explorer/) for the *C. elegans* embryo scRNA seq atlas developed by Packer et al. (2019).

## Data Availability

Description: FASTA files for the lim-8 final exon and knock-in sequences. Resource Type: Dataset. DOI: https://doi.org/10.22002/ffyv9-se005 Description: FASTA files for new alp-1 RNAi construct. Resource Type: Dataset. DOI: https://doi.org/10.22002/qgptf-s6f23
